# The Odonto-Chirurgical Society of Scotland

**Published:** 1886-02

**Authors:** 


ARTICLE II.

### THE ODONTO-CHIRURGICAL SOCIETY OF SCOTLAND.

The first meeting of the Session 1885-86 took place on the evening of November 12th, at the rooms of the Society, 30 Chambers street, Edinburgh, Mr. W. Bowman Macleod, L. D. S., Edinburgh, President, in the chair.

The President, in opening the Session, made a few remarks in acknowledgment of his election to the Presidential chair.

Mr. Wilson exhibited the upper models of two cases having relation to Dr. Edwards' paper.

In the first there were, on the right side, a central, a geminated lateral (two laterals,) and a canine, all temporary; on the left, a permanent central, with a lateral and a canine, both temporary. The central had succeeded a geminated tooth (central and lateral.)

In the second, the second premolar on the left side was represented by a rudimentary tooth whose crown was hemispherical. On the opposite side the temporary tooth was still in place.

Mr. Lipscomb made some observations upon a case of a gentleman, about forty years of age, who had called upon him, complaining of a nasty taste in his mouth, especially when he awoke in the morning. He had been troubled for about two years. He found that his health was suffering a good deal in consequence, and that he was getting weaker every day, this condition being, however, associated with comparatively no pain. He had consulted his doctor at intervals during the two years, but with no beneficial results. On examining his mouth, Mr. Lipscomb found his teeth in good order, but the upper left wisdom tooth missing. As the patient then stated that he did not think he had had a tooth extracted from that position, the cause of the trouble was very easily diagnosed, viz., that it was the discharge from a fissure in that region. A free incision was then made up to the alveolus, and plugged well with cotton wool saturated with a weak solution of carbolic acid. In a few days, the plug having been renewed, Mr. Lipscomb succeeded, after some considerable difficulty, in removing the tooth, the reasons of which difficulty would be very obvious to anyone examining the tooth, as the roots were largely exostosed, and there was the danger of breaking away the posterior wall of the superior maxilla to be borne in mind —a by no means impossible accident, considering the porous nature of the bone. The wound was dressed for a few days with solutions of chloride of zinc and carbolic acid alternately, and a tonic prescribed. He soon began to put on flesh and gain strength and has made a complete recovery.

Mr. Campbell said— About a year ago he extracted, after a good deal of trouble, an unerupted wisdom tooth, the details of which he thought worth mentioning in connection with Mr. Lipscomb's interesting case.

The patient, a lady aged 49, had suffered from neuralgia more or less severely, on the right side of her head and face, for five years. For two or three months before she came under his care her suffering had been great. She had consulted several medical men, but without relief. He (Mr.

Campbell) knew the lady's husband, who spoke to him casually about his wife's great suffering. He suggested an examination of her mouth, which proposal was acted upon, but the teeth appeared to be perfectly sound. He noticed, however, that the right upper wisdom tooth was missing, and from the fullness of the gum, did not seem to have been extracted. On enquiry, he found the patient had never had a tooth removed from the upper jaw. From the symptoms, and the appearance of the mouth in the region of the upper wisdom tooth, he came to the conclusion that this tooth was not erupted, and the probable cause of all the patient's suffering. After consultation with the family doctor, who happened to be an eminent surgeon, it was agreed to put her under chloroform, and examine the state of matters. This was done, and when the gum and alveolus had been cut through, the instrument came upon the enamel of the wisdom tooth. Feeling now fully assured of its presence, he proceeded to extract it, but never before experienced so much difficulty in removing a tooth from its socket. The tooth in front, and a part of the alveolus surrounding the wisdom tooth, which proved to be extremely dense, was removed, and even then it was with considerable difficulty that the tooth was at last extracted, it having to be literally gouged out. The tooth was slightly carious and considerably exostosed. The patient of course suffered a good deal for a week or two after the operation, but has since been quite free from pain.

The President then read the following paper, forwarded to him by Dr. H. H. Edwards, D. D. S., of Madrid, illustrated by nine drawings of models, and also a model of a supernumerary incisor carved in ivory :—

### THE MISSING INCISORS IN MAN. WHICH ARE THEY?

Gentlemen—In presenting the accompanying drawings to your excellent Society, I will; with your indulgent permission, supplement them with short descriptions of the same; also, if not trespassing too much on your valuable time—to say nothing of your patience—will endeavor, in as brief a manner as possible, to add a new argument to this highly interesting subject.

In the first place, allow me to publicly thank Mr. Wilson for having brought the subject to my notice, it being the publication of his able paper that set me thinking. Also, allow me to pay tribute to the courtesy of your present able President, for kindly arranging to bring my feeble effort before your society.

*Description of Cases Presented.*

No. 1 represents a mouth in which the six incisors evidently are intact. The canines were extracted when the patient was a youth, in order to correct an otherwise great inconvenience and deformity. The supernumerary teeth are those, one on either side of the median line. The one on the right side being a geminous tooth, the supernumerary half of which would naturally follow the central type.

No. 2. This case presents a supernumerary tooth erupted to the left of the median line, and extraordinarily well placed in the line of the arch. This tooth is, in all its appearances, a supernumerary tooth of the central type.

No. 3 is the model of a supernumerary tooth erupted to the right of the median line. This case is a common expression exhibited by supernumerary teeth, and requires no more mention than to say I believe it to follow the central type.

No. 4 presents a case which, with our present knowledge, we presume that the left side of the mouth is normal, but on the right side of the mouth there exists a space between the central incisor and the—as Prof. Turner puts it—pre-canine. This pre-canine evidently is a supernumerary tooth, small but partaking of both forms of central and canine; a sort of nondescript, which may or may not be a diminutive type of the, so put forward, missing outer-third incisor. But why—when, as in this case, there is a certain space left open—the lateral incisor is suppressed and this supernumerary tooth created, is a matter for discussion and investigation beyond my power

No. 5 is a somewhat similar case as the preceding one, with this exception, that the open space is between the lateral incisor and the canine on the right side of the mouth. In this case, the lateral incisor has erupted, and Nature seemed willing to leave space enough for the eruption of the outer-third incisor; but it was not forthcoming.

No. 6. This case is another exhibiting spaces left for the eruption of teeth, and the non-fulfilment of the same. This, if the centrals had been close together, would be a very common expression of the suppression of the lateral incisors. It is a question as to whether the spaces left between central incisors may or may not be intened by Nature for the eruption of teeth.

In No. 7 is exhibited an arrangement of the teeth, having the left lateral suppressed, and the canine well up to, and touching, the central incisor. I should also say that the right lateral incisor is suppressed; the pre-canine, though not clearly shown in the drawing, is the model it has the decided appearance of being—as in case 4—supernumerary.

No. 8 is a carved imitation in ivory of a specimen that came into my hands not long ago, having been extracted in order to clear the mouth for an artificial denture, and given me by the patient on account of its peculiarity. It is evidently a geminous lateral incisor. Here seems strong evidence in favor of the outer third incisor. I have never seen a similar tooth, and I believe it to be a rare and interesting specimen. In the original, a bristle can be passed into each pulp canal.

No. 9. It is but a few days since this case came to my notice, and I include it, as showing that the expression of No. 7 may appear on either side of the median line.

In cases Nos. 1 and 8 I have used the term "geminous tooth," and I believe I am right in doing so, though hardly in accordance with Prof. F. Flagg, Philadelphia Dental College., U. S. A. who says that "practically geminous teeth have but one pulp," and the cause of gemination is "abnormality of crown tissue." In case No. 1 it may be so, for we cannot diagnose a tooth in the mouth as possessing one or two pulps; but in case No. 8, although the crowns were joined during the process of creation, the roots are distinct, and each one possesses its separate pulp canal. They are not "attached teeth," for "though *they* have separate pulps their roots are attached to each other by the intervening wall of their alveoli; bone and cementum not uniting." Neither are they "fused teeth," that phase being brought about by exostosis or mal-position; consequently I hold to my right of calling them "geminous teeth."

It is perhaps needless to say I have cross-examined each of these patients—who are all educated, intelligent people—one of them being the present Home Secretary—to find out if any extractions had been done when young, for, I am sorry to say, there are dentists in this country who, through ignorance, are liable to extract a permanent tooth for a temporary one; but they all affirm that no such extractions have taken place.

I must apologise for the few cases presented, but I have presented them by selection, so as to cover the majority of variations that have come under my notice, which are many and variable; indeed, Nature seems to "ring the changes" upon the arrangement of these supernumerary teeth in a prolific manner.

Supernumerary teeth are undoubtedly presented to us by the great law of inheritance, the principal forms of which law may be divided into continuous, interrupted, collateral, and atavic.

The appearance of these supernumerary teeth is, I believe, due to the law of atavism; but upon whose authority we have it that pre-historic man possessed six incisors I know not, unless it be that of the "Evolutionist." I believe there are many who do not believe in the theory of evolution, but I will leave that question for more experienced men than I to argue. One observation I will make, though. I suppose that atavic characteristics appearing with comparative frequency is an effort on the part of Nature to bring to our notice forgotten facts—doubtless for our edification—though for our use is questionable. As in chemistry, our professors tell us that no matter what changes take place, no particle of matter is lost; so in Nature, to those who can read aright, no information is wanting, though seemingly lost, to instruct us in what existed pre-historically. She is full of signs and warnings, but there is a want of ability on the part of man to read her with sufficient intelligence.

There is an interesting article in the *Cosmos* for September, entitled, "Heredity and Development of the Teeth." by Dr. Alton Howard Thompson, which will bear careful reading. Although the "missing incisors in man" may or may not have been in his thoughts, I may be permitted to quote somewhat relating to that subject. He says, "The suppressive effects of disuse as affecting development of the teeth through heredity and variation are also to be noticed. In no class of organs are the inherited effects of variation due to the influence of changed conditions so marked as in teeth of man. Being thus susceptible to the effects of active employment or of neglect, they have, by the protracted operation of disuse weighing upon them and retarding their production for generations, become, as one of its effects, so defective and incomplete as to approach the condition of rudimentary organs. The active employment of an organ makes demand upon the nutritive powers for its growth and strength, which is responded to by increased nutrition and added strength by those powers; and use gives an impetus to transmission, which causes that organ to be well and strongly developed in the next generation. But disuse furnishes no stimulus to either nutrition or transmission, and the organ so affected is produced as tradition due to the stimulus of past generations, when it was in active employment, but owing to its disuse in recent generations, it is weak and ill-formed; it has not the necessary stimulus either for development or strength. Not only that, but an organ that has fallen into disuse and neglect becomes deleterious and injurious, and is, by a natural process of economy of growth, deprived of nutrition, that it may be suppressed and aborted. The remains of many such organs linger in the organization of man as rudiments of former organs which served a useful purpose under different modes of life: but the conditions of life being changed by new environments, these organs became useless, then injurious, and were gradually suppressed by the law of economy of growth. Such organs the teeth of man are rapidly becoming. Indeed the wisdom teeth have already arrived at that stage in their career of suppression when they are little more than rudiments. They are never well organized, are often rudimentary in form, and often totally absent, either through failure to erupt or development. The wisdom tooth in the race is departing, and we are the contemporary witnesses of the act of its abolition as a useless organ. Will the second molar follow it in time, and when the other teeth in more or less regular succession? We do not know, we only speak of what we observe. But we do know that all the teeth are defective in form, and deficient in structure, in most of the individuals of the luxurious races of man."

He does not appear to have noticed particularly the suppression of the lateral incisors, but only mentions that of the third molars. I will refer to the third molars later on.

Dr. Thompson also quotes M. Topinard (anthropology), who says, "The most remote ancestors have their share in forming the individuals, as well as the immediate parents. In atavism, the reappearance of character is a matter of chance, or rather there are in the germ latent powers, which are awakened into activity by favorable influences."

Also, he quotes Dr. Norman W. Kingsley, who says, "It is a most wonderful subject for contemplation, that at some remote period in the history of our progenitors, when Nature departed from the normal type, to produce, say, a deformed lateral incisor, a twisted cuspid, or to suppress a lateral, or a third molar, that following down the line of descent we find precisely the same peculiarity appearing and reappearing in the same line; and, again, not in the line, but in different branches of the family."

Of course if we ask for a reason why Nature interferes with existing laws to present us with a past specimen of her handiwork, we shall get no satisfactory response. In answer to an article of mine recently published in the *Dental Practitioner*, U. S. A. Dr. Dwight Ingersole says, among other good things, "The reason why Nature permitted physical laws to interfere with the laws of the organic kingdom has been asked for a thousand times, but no satisfactory answer has ever been given, because there is no reason for anything that has been brought into existence, the action of law. Nature seems to be blind and deaf to reason, and is arbitrary in all her ways. A reason was never given for anything until man came endowed with reason, and since his advent has seemed to be perfectly contented in allowing him to manifest the little he has, in his own way." I may add that there is great truth in the foregoing, notwithstanding its terseness.

Well, then, if Nature stands aside and leaves us to reason these things out, being content only to present us with the enigma, our duty evidently is to stand in the breach *a l'outrance*.

The question before us concerns the incisor teeth, but if I may be allowed to make a short digression, I would like to say a word about the molars. How many molar teeth did pre-historic man possess? Our nearest kinsfolk, I presume, by the law of evolution—if accepted—are the Simiadæ, of which there are three families, namely:—1, Arctopithecini; 2, Platyrrhini; and 3, Catarrhini. Now, these worthy kinsfolk—begging the pardon of any susceptible gentleman present—though their incsors are same in number as our own, they somewhat differ with regard to the number of their premolars and molars. No 1 possesses 3 pre-molars and 2 molars; No. 2 possesses 3 pre-molars and 3 molars; No. 3 possesses 2 pre-molars and 3 molars, on either side, above and below. I mention this for the following reason:—Some ten years ago I extracted a tooth—supernumerary of course—from the mouth of a woman in the west of England that had the appearance partly of a biscupid and partly of a molar, but very small. It had erupted buccally between the second and third molars on the right side of the upper jaw. As it is an undisputed fact that the third molar is being suppressed, and in everyday practice I see numbers that are little more than rudimentary, it may be that the third pre-molar has been suppressed. One case is not sufficient to prove anything, but if there are other gentlemen who have met with similar cases, I would like very much to know their opinion. Certainly it would strengthen materially the theory—if so be there is a theory—of the suppression of outer thirds, namely; —The third molar, the third pre-molar, and the third incisor. Whoever has noticed the jambing and difficult eruption of the lower third molars that so frequently takes place, must also have remarked a shortening of the jaw itself, at its posterior portion. The law of economy of growth will not admit of superfluous organs, or parts of organs; therefore, if the third molars are to be suppressed. the jaws will shorten in consequence; or if the jaws take the initiative, then the molars must be crowded out.

Now let us return to the incisors. If so be that prehistoric man possessed six incisors, the question is—which pair or pairs have been suppressed?

At the outset, I think it is of vital interest that the period of eruption of the supernumerary teeth should be carefully recorded. I have never been fortunate enough to see a supernumerary tooth during the process of eruption; it would doubtless be of use in ascertaining to which type it belonged, and serve as useful data, so that we might reasonably fix the succession in which it originally erupted. If, by the law of atavism, the suppressed tooth appears, its appearance, by the same great law, should take place in its original succession. We know that the central incisors erupt at the age of 6-8 years; the lateral incisors, 7-9 years; and the first biscuspids from 9-10 years. Therefore, if the supernumerary tooth be of the lateral type, it should erupt between the ages of 8-10 years; if, on the contrary, it be of the central type, according to the position in which we place it, namely, as a suppressed *central*, it should erupt between 5 and 6 years of age; while, if a lateral following the central type, between the ages of 6 and 8 years.

I have no doubt that some gentlemen will take exception to my fixing a date for the eruption of supernumery teeth, and may with fairness say, that if in form these teeth are erratic, their eruption may also be erratic, and therefore, cannot form a reliable basis.

And now for a "new departure." Since having my attention drawn to this subject by Mr. Wilson's paper, I have differed somewhat in my mind from his views, and am more inclined to look upon the supernumerary tooth as being a rudimentary *central.*

The incisors are the teeth of prehension, and the centrals naturally are the most prehensile; therefore, if suppression has taken place through disuse, I infer that the original centrals would be the first to disappear. That the present lateral incisors are *now* being suppressed is not denied. But why? Because the present centrals still find sufficient employment to warrant their continuance, whilst the laterals are becoming more and more superfluous. In about 25 per cent. of my patients, I find the central incisors separated, while the lateral incisors of 50 per cent. possess cutting edges inclining more towards the canine arc than following the central cutting line. As a curious coincidence, whether arising from observation or professional direction, the manufacturers of artificial teeth are making their lateral incisors every day more and more like unto the happy medium between centrals and canines, namely, with rounded corners; they profess to have made them from impressions taken from the natural teeth, and in practice, whether for pivoting or plate making, that is the class of tooth I mostly use. It may possibly be a peculiarity possessed by the Spaniards, but, as I say, I find a goodly per centage with spaces between the centrals, the centrals small in comparison with the canines and molars, and the laterals possessing the intermediate form between centrals and canines, even to the labial longitudinal ridge. The above observations being forced upon me daily, with an occasional supernumerary tooth or teeth in the medium line or space, as in cases Nos. 1, 2, and perhaps, 3, make me strongly inclined toward the idea, namely, the suppression of the original *centrals* from generations of disuse.

Of course this is only surmising, but I would present the idea, with a view of widening the field of vision. We know that a solution sought for is invariably found, provided we wish that particular solution to *be* found ; if a man wants a particular cause in order to explain a particular lesion, he will generally adopt that one which appears right to him and work hard at his proofs, not deigning to accept any innovations. It is partly with the idea of aborting such a catastrophe that I have presented the possibility of the supernumerary tooth or teeth as being suppressed *centrals.*

If the idea—mind, I assert nothing—brings forth good fruit in the shape of a thoroughly useful discussion of the subject I shall be satisified ; often a dissenting voice will either clench the accepted idea, or it will set men thinking of other possibilities.

On such meagre evidence as we possess, it is the wildest folly to found a theory ; all we can do is to collect facts that will enable the next genenation to form a firm basis. Facts, gentlemen, facts ! Let all collect, accumulate, and register them in such quantities, that when *we* are gone there may be no excuse for the coming physiologist to taunt us with idleness or indifference.

In conclusion, gentlemen, I would earnestly impress upon all professional men, especially those whose practice commands the care of children's teeth, as in dental hospitals, to watch the development of these supernumerary teeth, and to carefully record all such cases that come under their notice.

As the paper was one which required some amount of thought it was considered better to postpone the discussion upon it until the next meeting, by which time the paper would be in the hands of the members ; and it was decided to have woodcuts made of seven of the more important illustrations for the better consideration and study of the cases in question.

The President then proposed that Dr. Edwards should be elected as a corresponding member of the Society, and that a vote of thanks should be accorded to him for his labour and trouble he had been to in order to bring the matter under their notice, both of which propositions were carried *nem. con.*; and the Secretary instructed to communicate the same to Dr. Edwards.—*London Dental Journal.*

---

ARTICLE III.

